# Silicon deposition in nanopores using a liquid precursor

**DOI:** 10.1038/srep37689

**Published:** 2016-11-22

**Authors:** Takashi Masuda, Narihito Tatsuda, Kazuhisa Yano, Tatsuya Shimoda

**Affiliations:** 1 School of Materials Science, Japan Advanced Institute of Science and Technology, 1-1 Asahidai, Nomi, Ishikawa 923-1292, Japan; 2 Toyota Central R&D Labs. Inc., 41-1 Yokomichi, Nagakute, Aichi 480-1192, Japan

## Abstract

Techniques for depositing silicon into nanosized spaces are vital for the further scaling down of next-generation devices in the semiconductor industry. In this study, we filled silicon into 3.5-nm-diameter nanopores with an aspect ratio of 70 by exploiting thermodynamic behaviour based on the van der Waals energy of vaporized cyclopentasilane (CPS). We originally synthesized CPS as a liquid precursor for semiconducting silicon. Here we used CPS as a gas source in thermal chemical vapour deposition under atmospheric pressure because vaporized CPS can fill nanopores spontaneously. Our estimation of the free energy of CPS based on Lifshitz van der Waals theory clarified the filling mechanism, where CPS vapour in the nanopores readily undergoes capillary condensation because of its large molar volume compared to those of other vapours such as water, toluene, silane, and disilane. Consequently, a liquid-specific feature was observed during the deposition process; specifically, condensed CPS penetrated into the nanopores spontaneously via capillary force. The CPS that filled the nanopores was then transformed into solid silicon by thermal decomposition at 400 °C. The developed method is expected to be used as a nanoscale silicon filling technology, which is critical for the fabrication of future quantum scale silicon devices.

The constant scaling down of device dimensions through state-of-the-art microfabrication techniques has driven the continuous growth of the semiconductor industry. However, huge required capital outlays and physical processing limits of fine patterning are recognized as urgent issues. A shift from two-dimensional (2D) planar structures to three-dimensional (3D) vertical structures has been proposed as a solution to these scaling challenges^1^.

Embedding of pores with silicon is a key technology for the fabrication of 3D structures. In the field of 3D packaging, the through-silicon via (TSV) approach satisfies the constraints of high interconnection density and high data throughput, in conjunction with good signal integrity^2^. In the field of flash memory, the stacking of cells in the vertical direction instead of the shrinking of cells within a 2D plane overcomes the capacity limitation of traditional 2D cells. In this approach, pores with a diameter of approximately 50 nm are formed in a 48-layer stacked cell, and the inside walls of the pores are coated with silicon dioxide, silicon, or other materials that impart capacitance^3^,^4^.

The most widespread techniques for embedding materials into pores are sputtering and chemical vapour deposition (CVD). However, an intrinsic shortcoming of these techniques is their limited ability to coat the inside wall of pores. Films deposit onto the surface, closing the entrance of the pores and resulting in thinner films deep within the pores. Tapered pores^5^ and the seed-layer enhancement technique^6^ have been proposed as solutions to this problem. Although these techniques are currently effective, they would not be easily adapted to further scaling down of the pores (i.e. to the single-nanometer scale).

In this study, we demonstrate a method to deposit silicon onto the inner wall of pores with single-nanometer-scale (3.5 nm) openings and a high aspect ratio (70). We synthesized liquid cyclopentasilane (CPS) and deposited it via liquid-source vapour deposition (LVD). We originally developed CPS as a liquid precursor for semiconducting silicon^7^, and it has been used for solution processing of silicon devices^8^,^9^,^10^. Moreover, vaporized CPS has been reported to be a good gas source in LVD^11^. LVD is a thermal-CVD method conducted under atmospheric pressure, in which liquid CPS was placed in a deposition chamber and was vaporized by heating to generate a gas source^12^.

Here, we report our discovery that silicon can be deposited deep into the nanopores of monodispersed mesoporous carbon sphere (MMCS) by LVD using CPS. LVD fills the nanopores with CPS, which is subsequently transformed into solid silicon by thermal decomposition at 400 °C. An important feature of CPS is its low vapour pressure and high cohesion energy, which are attributed to its high molar mass. Thus, the deposition mechanism of CPS is expected to differ from that of conventional CVD sources such as silane and disilane. The purpose of this study is to estimate the free energy of CPS introduced into nanopores and to clarify the filling mechanism of the nanopores. The ability to form silicon in nanopores is critical for the fabrication of 3D stacked or quantum scale devices. LVD using CPS has the potential to replace conventional sputtering and CVD processes in the semiconductor field as devices are downscaled further.

## Results and Discussion

### Characterization of Si-MMCS

**Figure 1:**
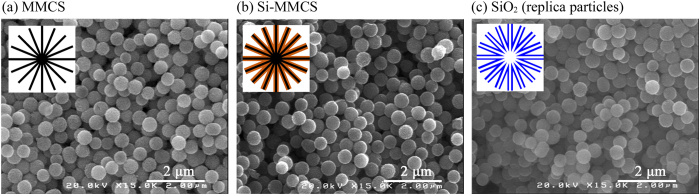
SEM images of obtained particles: (**a**) MMCS; (**b**) Si-MMCS; and (**c**) Si-MMCS after calcining in air at 1000 °C, which decomposed the carbon component, leaving replica particles composed of SiO_2_. A schematic is inserted in the upper left corner of each SEM image.

We observed the appearance of MMCS before and after LVD. Figure 1(a,b) show the scanning electron microscopy (SEM) images of MMCS and Si-MMCS, respectively, in which Si-MMCS is a composite material of silicon and MMCS obtained via LVD. The diameter of MMCS was 500 nm and was unchanged after LVD, as shown in Fig. 1(b). In contrast, the weight increased from 0.20 g for MMCS to 0.31 g for Si-MMCS. Silicon was likely deposited inside the MMCS nanopores. Internal silicon was confirmed by the removal of the carbon template. Figure 1(c) shows a SEM image of a Si-MMCS sample calcined in air at 1000 °C; the calcining procedure decomposed the carbon component, and replica particles composed of SiO_2_ were formed. The SiO_2_ particles were similar in size to the MMCS particles before calcining. These SEM images reveal that CPS vapour penetrates into the nanopores and transforms into solid silicon. A schematic depiction of each particle shape is inserted in each image.

**Figure 2:**
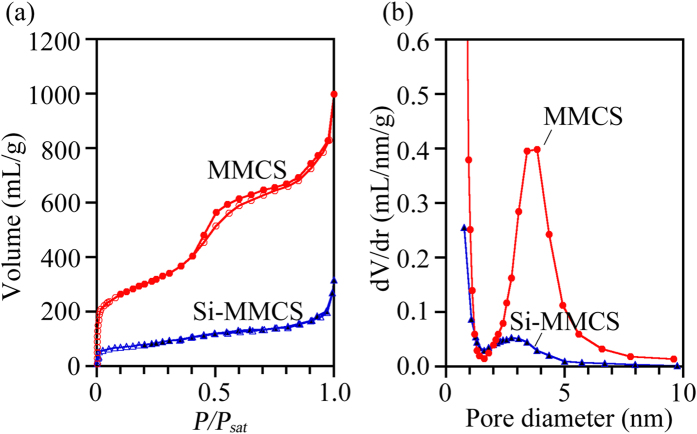
(**a**) Adsorption–desorption isotherms of MMCS and Si-MMCS at 77 K; open and closed symbols correspond to adsorption and desorption, respectively. (**b**) Pore size distributions of MMCS and Si-MMCS. Major peaks appeared at 3.5 nm (MMCS) and 2.7 nm (Si-MMCS).

Figure 2(a) shows the MMCS and Si-MMCS nitrogen adsorption–desorption isotherms collected at 77 K. MMCS exhibited a number of adsorption events in the initial stage because the nanopores contained tiny slaps. The adsorption amount in the entire area as well as in the initial stage was reduced in Si-MMCS because of silicon filling. The desorption branch for MMCS showed a small decrease at 0.4 ≤ *P*/*P*_sat_ ≤ 0.5, corresponding to nanopores with a diameter of 3.5 nm. The pore size distribution (PSD) plots were obtained from the adsorption branch of the isotherms using the Barrett–Joyner–Halenda method, which is based on the assumption of cylindrical pore geometry. Figure 2(b) shows the PSD plots of MMCS and Si-MMCS. As silicon was deposited into MMCS, the peak pore diameter shifted from 3.5 nm (MMCS) to 2.7 nm (Si-MMCS) and the peak intensity decreased.

**Figure 3:**
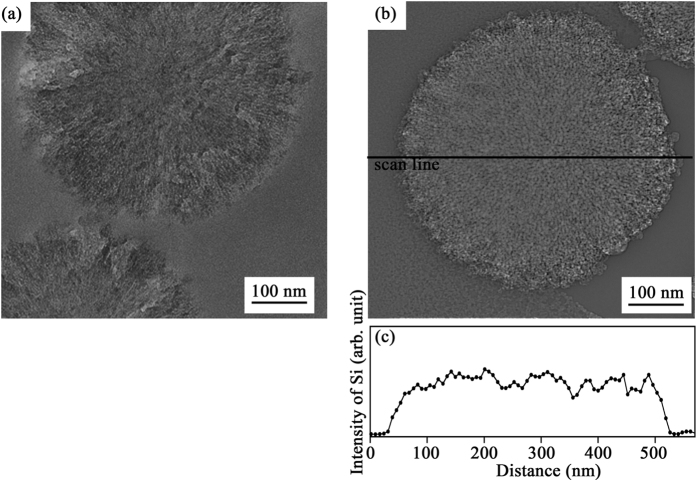
Cross-sectional TEM image of a sliced particle: (**a**) MMCS and (**b**) Si-MMCS. (**c**) The concentration of silicon analysed by line-scan EDX. Scanning line of the EDX is drawn in Fig. 3(b). The intensity associated with silicon is plotted.

We performed cross-sectional transmission electron microscopy (TEM) observations to directly observe the interior of MMCS and Si-MMCS, as shown in Fig. 3(a) and (b), respectively. Line analysis using energy-dispersive X-ray (EDX) spectroscopy was also performed to confirm the presence of internal silicon in Si-MMCS, as shown in Fig. 3(c). Both the particles were sliced into thin disk. Radial patterns with a spacing of several nanometers between two neighbour lines near the edge were observed in both MMCS and Si-MMCS particles, whose dimension corresponded to the diameter of the nanopores. The EDX intensity of silicon was uniform inside the Si-MMCS particle, as shown in Fig. 3(c). These results of our experiment clearly show that CPS penetrated deep into the nanopores.

We here speculate on the validity of the amount of silicon deposited in Si-MMCS. In our experiment, the weight increased from 0.20 g (MMCS) to 0.31 g (Si-MMCS) after LVD. Thus, 0.11 g of solid silicon was deposited in the MMCS. MMCS (0.20 g) had a total pore volume of 0.24 mL (1.217 mL/g). If we assume that CPS vapour condensed to the liquid state in the nanopores, then the maximum weight of CPS would be 0.23 g (CPS = 0.96 g/mL^13^). Hence, in our case, the filling rate of silicon was 48 wt%.

The thermal properties of silicon hydride compounds have been reported in the literature^14^; this material transformed into silicon, accompanied by the release of a large amount of SiH_4_/H_2_ gases, at temperatures above 120 °C and stabilized at 400 °C as amorphous silicon. During the conversion process, the material lost 58% of its weight when heated at 400 °C and only 42 wt% of the material remained as silicon, consistent with our value of 48 wt%. Presumably, the nanopores were filled with liquid CPS during the initial stage of LVD and the CPS-to-silicon conversion was subsequently induced, resulting in a weight reduction of 50–60%. The validity of this hypothesis is discussed in the next section.

### Theoretical evaluation of capillary filling in MMCS

In this section, we estimate the disjoining pressure *Π* of CPS based on Lifshitz van der Waals (vdW) theory and consider the adsorption mechanism of CPS onto the inner wall of MMCS. Then, we speculate the filling mechanism of the nanopores.

**Figure 4:**
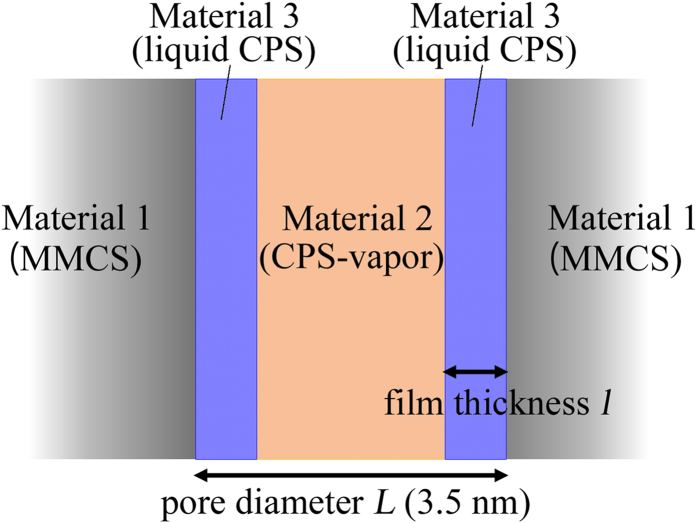
Schematic of the adsorption process of liquid CPS on the inner wall of MMCS during LVD.

During the LVD process, CPS vapour adsorbs onto the inner wall as a liquid film in proportion to its vapour pressure. The simplest case considered here is that of parallel plates covered with only a thin film of liquid CPS (Fig. 4). The presence of the opposing liquid film modifies Supplementary Eq. (S.2). The relationship between parallel plates and distance (pore diameter) *L*, equilibrium liquid film thickness *l*, and *Π* is expressed as follows^15^:


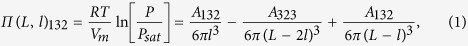


where *R, T, V*_*m*_, and *P/P*_sat_ are the gas constant, absolute temperature, molar volume, and relative vapour pressure, respectively. *A*_132_ and *A*_323_ are the Hamaker constants for solid–vapour interactions through intervening liquid and for liquid–liquid interactions through intervening vapour, respectively. The derivation of *A* is described in the Supplementary Information. The value of *A*_132_ typically lies between 1 and 100 zJ and is negative when the liquid wets the solid. The liquid film stability was affected by the vdW interaction of the opposite side through the narrow gap.

Before estimating *Π* directly from Eq. (1), we speculate on the mechanism of the evolution from CPS vapour to a liquid layer, which is associated with capillary condensation. Capillary condensation is associated with a phase transition related to the coexistence of vapour and liquid in the pores. A vapour confined in a pore condenses at a pressure lower than the saturation vapour pressure of the corresponding bulk liquid because the condensed state is stabilized by the strong attractive interactions between vapour molecules and pore walls. As a result, capillary condensation induces the filling of nanopores with liquid.

At a certain critical film thickness *l*_c_ or critical *P/P*_sat_, the liquid film loses its stability and coalesces, followed by a spontaneous filling of the pore^16^. This phase transition from dilute vapour to a dense liquid is the aforementioned process of capillary condensation. Capillary condensation arises at ∂*Π*/∂*l* = 0^17^,^18^, such that


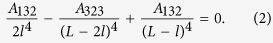


The last term in Eq. (2) is neglected as a first approximation. *l*_c_ at the point of capillary condensation is given by


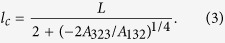


By substituting the obtained value of *l*_c_ into Eq. (1), we obtain the critical *P/P*_sat_ at the point of capillary condensation. These calculated thermodynamic values are summarized in Table 1. The values for water and toluene are also listed for reference. A process temperature of *T* = 393 K was used in the calculations.

The larger negative value of *A*_132_ for CPS compared to those for water and toluene indicates a stronger interaction between CPS and MMCS; specifically, liquid CPS wets MMCS well. Indeed, liquid CPS, when dropped onto MMCS directly, penetrates into the nanopores immediately^19^. Similarly, the larger value of *A*_323_ for CPS compared to those for water and toluene arises from the greater cohesion energy of CPS, which, in turn, stems from its larger molar mass and higher refractive index (see Supplementary Table S1). Nonetheless, the *A* values for CPS, water, and toluene are all similar to each other. Consequently, they exhibit similar values of *Π* and *l*_c_. In the case of the critical *P/P*_sat_ at the point of capillary condensation, CPS shows a *P/P*_sat_ as small as 0.76, whereas water and toluene show *P/P*_sat_ values of 0.97 and 0.85, respectively. The small critical *P/P*_sat_ value of CPS stems from its larger *V*_*m*_ (see Supplementary Table S1, *V*_*m*_ = molar mass/density) in Eq. (1); specifically, CPS tends to exhibit capillary condensation at lower *P/P*_sat_ values compared to water and toluene because of its larger molar mass. CPS vapour that has been introduced into nanopores is expected to induce capillary condensation more easily than other materials during the LVD process. We note that the critical *P/P*_sat_ values of conventionally used CVD sources such as silane and disilane should be large because of their small molar masses, indicating that they will not easily undergo capillary condensation during the deposition process.

**Figure 5:**
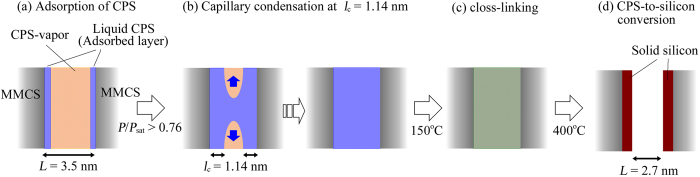
Proposed model for silicon deposition in nanopores during LVD: (**a**) vaporized CPS adsorbs onto the inner wall of nanopores; (**b**) the liquid film loses its stability and coalesces, followed by a spontaneous filling of the pore at *P/P*_sat_ > 0.76 or *l*_c_ > 1.14 nm; (**c**) condensed CPS solidifies via a cross-linkage reaction at 150 °C; (**d**) the cross-linked CPS transforms into solid silicon at 400 °C, accompanied by a one-fourth reduction in volume.

The silicon deposition process based on our results is summarized in Fig. 5. CPS vapour adsorbs onto the inner wall as a liquid film in proportion to its vapour pressure (Fig. 5(a)). As mentioned in the Methods section, a sufficient quantity of CPS to realize *P/P*_sat_ > 0.76 or *l*_c_ > 1.14 nm was placed in the sample chamber. Therefore, CPS vapour condenses into liquid CPS in the nanopores via capillary condensation. The two films form a liquid bridge followed by a spontaneous filling up of the space (Fig. 5(b)). Subsequently, condensed CPS solidifies via a cross-linkage reaction at 150 °C (Fig. 5(c)). Because the cross-linking temperature (>120 °C)^20^ overlaps with the temperature at the point of capillary condensation (120 °C), the condensed CPS would turn into gel before capillary evaporation. Finally, the cross-linked CPS transforms into solid silicon at 400 °C^14^ (Fig. 5(d)). CPS-to-silicon conversion results in volume shrinkage as a result of the change in density from 0.96 g/cm^3^ (CPS) to 2.2 g/cm^3^ (silicon^21^). Moreover, as previously mentioned, a weight reduction of approximately 50–60% also occurs during the conversion^14^. As a result, the volume of CPS is reduced by one-fourth and narrower pores (2.7 nm) appear in preexisting nanopores (3.5 nm), as shown in Fig. 2(b).

## Conclusion

As the sizes of pores decrease, surface effects become increasingly important. In this study, we focused on capillary condensation, which results from the effect of surfaces on the phase diagram of a fluid and is a ubiquitous phenomenon at the nanoscale. We estimated the free energy in the system from the viewpoint of vdW interaction, and clarified that vaporized CPS induces capillary condensation much more easily than other materials such as water, toluene, silane, and disilane because CPS has a larger molar volume. During the LVD process, CPS vapour was transformed into solid silicon in the nanopores via the liquid state. Therefore, liquid-specific effects were observed during the deposition process; specifically, condensed CPS penetrated into nanopores spontaneously by capillary force. The LVD of CPS fundamentally differs from the CVD of silane/disilane in that capillary condensation occurs. The aforementioned liquid-specific feature in the LVD process is a unique and attractive method for nanoscale silicon deposition. Indeed, we realized silicon deposition deep into nanopores with openings 3.5 nm in diameter and an aspect ratio of 70 in this study. The combination of CPS and LVD is a promising technology for next-generation nanoscale deposition in the silicon industry.

## Methods

### Deposition procedure

**Figure 6:**
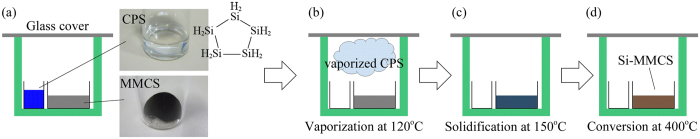
Schematic of the LVD process using CPS: (**a**) CPS and MMCS are placed in a deposition chamber away from each other; (**b**) CPS is vaporized at 120 °C; (**c**) CPS adsorbed onto MMCS solidifies via a cross-linking reaction at 150 °C; and (**d**) cross-linked CPS is transformed into solid silicon via thermal decomposition at 400 °C.

The advantage of LVD as a methodology stems from the attributes of CPS. When used as a gas source, CPS does not require gas bombs or carrier tubes for its containment and use because it is a liquid. Silicon gas can be locally generated anywhere in a deposition chamber through appropriate placement of liquid CPS. These features, combined with the potential for nonvacuum processing, simplify equipment design, as shown in Fig. 6. The cylinder-type chamber was constructed of stainless steel and had a volume of 27 cm^3^ (diameter of 25 mm × height of 55 mm). Because CPS is pyrophoric, all of the procedures were carried out in a glove box filled with nitrogen gas. The oxygen concentration and the dew point in the glove box were less than 0.5 ppm and less than −75 °C, respectively.

MMCS and CPS were synthesized according to the procedure described in our previous study^22^,^23^. MMCS have nanopores with a diameter of 3.5 nm and a pore volume of 1.217 mL/g, as previously discussed. MMCS (0.20 g) and CPS (0.73 g) were placed in the chamber apart from each other. A glass plate was then placed on the chamber as a cover. The volume of CPS was three times greater than the pore volume of MMCS.

The chamber was heated on a hotplate at 120 °C for 10 min to vaporize CPS; 70% of CPS was vaporized, and 30% of it was remained as thermally polymerized CPS (see Supplementary Figure S2). The volume of vaporized CPS reached 110 cm^3^, as calculated using the ideal gas law; the volume of the deposition chamber was 27 cm^3^. Because excess CPS vapour leaked out by pushing up the glass cover, internal pressure remained at approximately 1 atm. The chamber temperature was subsequently increased to 150 °C, and this temperature was maintained for 10 min to solidify adsorbed CPS on the MMCS. Cross-linking due to the 1,2-hydrogen shift reaction led to solidification of CPS; this cross-linking process is known to occur in silicon hydride compounds when the temperature exceeds 120 °C^20^. Finally, the chamber was heated at 400 °C for 30 min to complete the transformation from CPS to solid silicon. Composite particles (Si-MMCS) were synthesized in the chamber.

### Characterization

MMCS and Si-MMCS were characterized by SEM, thermogravimetric analysis (TGA), and TEM. The vaporization characteristics of CPS are important because CPS vapour was used as a gas source in this study. The thermal properties of liquid CPS were measured using TGA (Seiko Instruments EXTAR-6200) under a nitrogen atmosphere, where CPS was heated from 30 °C to 400 °C at a heating rate of 5 °C/min. A Hitachi S-4100 scanning electron microscope was used to analyze the surface and diameter of the particles. The quantity of silicon in Si-MMCS was estimated from the change in weight of the MMCS sample before and after silicon deposition. The pore sizes and volumes of samples were estimated on the basis of nitrogen adsorption–desorption isotherms collected at 77 K using a BELSORP-mini II (MicrotracBEL Corp). The samples were evacuated at 150 °C under 0.13 Pa before the measurement. Cross-sectional TEM images of the particles were obtained using a JEM-ARM200F (JEOL Ltd).

## Additional Information

**How to cite this article**: Masuda, T. *et al*. Silicon deposition in nanopores using a liquid precursor. *Sci. Rep.* **6**, 37689; doi: 10.1038/srep37689 (2016).

**Publisher’s note:** Springer Nature remains neutral with regard to jurisdictional claims in published maps and institutional affiliations.

## Supplementary Material

Supplementary Information

## Figures and Tables

**Table 1:** Calculated Hamaker constants *A*_132_ and *A*_323_, critical film thickness *l*_c_ at the point of capillary condensation, disjoining pressure *Π*, and critical relative vapour pressure *P/P*_sat_ at the point of capillary condensation.

Liquid film	CPS	Water	Toluene
*A*_132_ (zJ)	−98.7	−89.6	−94.6
*A*_323_ (zJ)	66.7	42.8	47.5
*l*_c_ (nm)	1.14	1.17	1.17
*Π* (Pa)	−5.88 × 10^6^	−4.80 × 10^6^	−5.14 × 10^6^
Critical *P/P*_sat_	0.76	0.97	0.85
