# Supplementary material for: Silicon deposition in nanopores using a liquid precursor
Source: Sci Rep. 2016 Nov 22;6:37689. doi: 10.1038/srep37689 (PMC5118725; doi:10.1038/srep37689)
Supplement: Supplementary Information [file srep37689-s1.pdf]

Supplementary Information

Silicon deposition in nanopores using a liquid precursor

Takashi Masuda[1,*], Narihito Tatsuda[2], Kazuhisa Yano[2], Tatsuya Shimoda[1]

[1] School of Materials Science, Japan Advanced Institute of Science and Technology, 1-1 Asahidai, Nomi, Ishikawa 923-1292, Japan

[2] Toyota Central R&D Labs. Inc., 41-1 Yokomichi, Nagakute, Aichi 480-1192, Japan

Estimation of free energy. The simple model considered here is that of a liquid thin film on a solid surface because CPS vapour was adsorbed onto the solid surface during the LVD process. Interfacial interactions resulted in a difference in chemical potentials between the liquid in the adsorbed film and the bulk phase. This difference in chemical potential is expressed as Π[24], which is the equivalent interfacial force per unit area of the interface and is a function of film thickness l. Parameter Π is associated with Gibbs free energy G as follows[25,26]:

$$\Pi = -\frac{\partial G}{\partial l}. \qquad \text{(S.1)}$$

The use of Π has advantages in cases where G is difficult to define, such as when interfacial zones overlap to the extent that the film does not retain the intensive properties of the bulk phase. From an experimental viewpoint, different interactions (e.g. vdW, acid–base[27], electrostatic interactions, and structural effects) are more easily accounted for with Π than with G. Among such interactions, vdW interactions are always present and substantially influence physical phenomena at the nanoscale in systems of nonaqueous/nonpolar materials such as CPS and carbon[28].

Given the domination of vdW interactions, Π is expressed using Hamaker constant A. The derivation of A is described in the next section. The $\Pi(l)_{132}$ per unit area acting between material 1 and 2 through intervening material 3 with distance l is represented using Hamaker constant A_{132} as follows[29]:

$$\Pi = \frac{A_{132}}{6\pi l^3}. \qquad \text{(S.2)}$$

Thermodynamic parameters are associated with P/P_{sat} through the Kelvin equation[30]:

$$\Pi = \frac{RT}{V_m} ln\left[\frac{P}{P_{sat}}\right]. \qquad \text{(S.3)}$$

Derivation of the Hamaker constant. Nonretarded Hamaker constant A_{132} is written as Eq. (S.4)[31,32]:

$$A_{132} = \frac{3k_BT}{2}\sum_{m=0}^{\infty}{}'\sum_{s=1}^{\infty}\frac{(\Delta_{13}\Delta_{23})^s}{s^3}, \qquad \text{(S.4)}$$

$$\Delta_{kj} = \frac{\varepsilon_k(i\xi_m)-\varepsilon_j(i\xi_m)}{\varepsilon_k(i\xi_m)+\varepsilon_j(i\xi_m)}. \qquad \text{(S.5)}$$

The primed summation indicates that only one-half of the first term in the frequency sum is counted. The summation is understood to include only terms that satisfy the sampling interval $\xi_m = m(2\pi k_BT/\hbar)$, where $\hbar$ and k_B are Planck's constant and the Boltzmann constant, respectively.

Plots of $\varepsilon(i\xi_m)$ vs. ξ_m, which we referred to as London-dispersion (LD) spectra, were used to estimate A_{132} values. The spectra of $\varepsilon(i\xi_m)$ were calculated from the imaginary part of the dielectric function $\varepsilon(\omega)$ by mathematical manipulation; $\varepsilon(\omega)$ is represented using real and imaginary parts denoted by prime (′) and double prime (″), respectively, as follows:

$\varepsilon(\omega) = \varepsilon'(\omega) + i\varepsilon''(\omega)$. (S.6)

Each part is related to refractive index $n(\omega)$ and absorption coefficient $k(\omega)$ via the following relations[33]:

$\varepsilon'(\omega) = n^2(\omega) - k^2(\omega)$, (S.7)

$\varepsilon''(\omega) = 2n(\omega)k(\omega)$. (S.8)

The real, ε' and n, and imaginary, ε'' and k, parts are related through the Kramers–Kronig (K–K) relation. The quantity $\varepsilon(i\xi)$ used in Eq. (S.5) is defined using the K–K relation:

$$\varepsilon(i\xi) = 1 + \frac{2}{\pi}\int_0^{\infty}\frac{x\varepsilon''(x)}{x^2+\xi^2}dx. \qquad \text{(S.9)}$$

When the $\varepsilon''(\omega)$ data are not available for a wide range of ω, the data are usually interpolated using a model that treats the material response to an electric field as a series of damped harmonic oscillators. The following Ninham–Parsegian representation is the well-known model used to estimate $\varepsilon(i\xi)$[34]:

$$\varepsilon(i\xi_m) = 1 + \frac{C_{IR}}{1+(\xi_m/\omega_{IR})^2} + \frac{C_{UV}}{1+(\xi_m/\omega_{UV})^2}, \qquad \text{(S.10)}$$

$$C_i = \frac{2}{\pi}\frac{f_i}{\omega_i}. \qquad \text{(S.11)}$$

In Eq. (S.10), $\varepsilon(i\xi_m)$ is described by the parametric Lorentz oscillator model with a resonance frequency ω_i and an oscillator strength f_i. The indices (IR and UV) for ω_i and C_i represent the values in the infrared and ultraviolet regions, respectively. These values for transparent materials were determined by a Cauchy plot of refractive index measured using an Abbe refractometer and ellipsometry. The physical and optical parameters used in this study have been described in our previous study[35)] and are summarized in Table S1.

Table S1. Physical and optical properties of CPS, water, and toluene. Optical parameters are quoted from our previous study[35)].

	CPS	Water	Toluene
Molar mass (kg/mol)	0.15	0.018	0.092
Dielectric constant ε	2.85	80.3	2.24
Refractive index n at 589 nm	1.6951	1.3333	1.4969
Density at 293 K (kg/m^3)	963	1000	867
Molar volume V_m (m^3/mol)	1.56×10^{-4}	1.80×10^{-5}	1.06×10^{-4}
Resonance frequency ω_{IR} (rad/s)	1.66×10^{14}	5.66×10^{14}	1.37×10^{14}
Resonance frequency ω_{UV} (rad/s)	1.13×10^{16}	1.86×10^{16}	1.38×10^{16}
Function of oscillator strength C_{IR}	0.1256	3.4222	0.0669
Function of oscillator strength C_{UV}	1.7244	0.7527	1.1731

In this study, LD spectra for CPS and MMCS were obtained using the difference route. $\varepsilon(i\xi_m)$ for CPS, water, and toluene were calculated via Eq. (S.10) using the measured values of ω_i and C_i, whereas that for MMCS was calculated via Eqs. (S.7)–(S.9) using *nk* data for carbon[36)]. The *nk* data are shown in Fig. S1(a), and the LD spectra of CPS, water, toluene, and MMCS at T = 393 K are shown in Fig. S1(b). The thin lines connecting the symbols are guides for the eye.

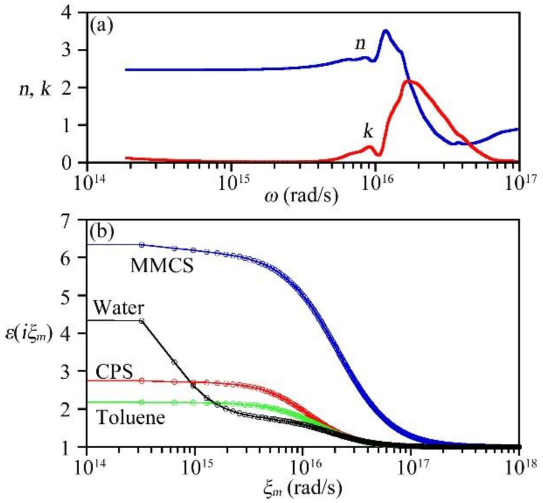

Figure S1. (a) *nk* data of carbon. (b) LD spectra of CPS, water, toluene, and MMCS (T = 393 K).

Thermal properties of CPS. The evaporation characteristics of CPS were measured by TGA. The thermogravimetry (TG) and differential thermogravimetry (DTG) curves are plotted as functions of the heating temperature in Fig. S2. Seventy percent of the mass readily evaporated at temperatures below 140°C. TG–mass spectrometry clarified that the vaporized component was attributable to CPS molecules. The remaining 30% of the mass gradually evaporated between 140°C and 360°C, which we attributed to thermally polymerized silicon hydride. The polymerization temperature (120°C~) is close to vaporized temperature (~140°C). In the DTG curve, major vaporization was observed at 120°C. Therefore, we used 120°C as the vaporized temperature for CPS.

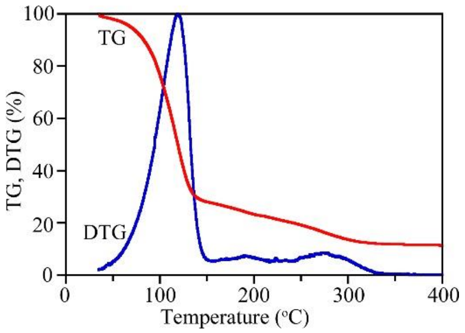

Figure S2. TG and TGA curves of CPS. Significant weight loss was observed at 120°C.

References

[24] Derjaguin, B. V. Correct form of the equation of capillary condensation in porous bodies. Proc in Int. Cong. of Surface Activity, **2**, 153–159 (Butterworths Scientific Publications, 1957).

[25] Derjaguin, B. V., Churaev, N. V. & Muller, V. M., *Surface forces*, 25–52 (Springer US, 1987).

[26] Nitao, J. J. & Bear, J. Potentials and their role in transport in porous media. *Water Resour. Res.* **32**, 225–250 (1996).

[27] Oss, C. J. V., Chaudhury, M. K. & Good, R. J. Interfacial Lifshitz-van der Waals and polar interactions in macroscopic systems. *Chem. Rev.* 88, 927–941 (1988).

[28] Masuda, T., Matsuki, Y. & Shimoda, T. Stability of polydihydrosilane liquid films on solid substrate, *Thin Solid Films* **520**, 5091–5096 (2012).

[29] Iwamatsu, M. & Horii, K. Capillary condensation and adhesion of two wetter surfaces. *J. Colloid Interface Sci.* **182**, 400–406 (1996).

[30] Adamson, A. W. *Physical Chemistry of surface*, 5th ed. (John Wiley & Sons Inc, 1990).

[31] Hough, D. B. & White, L. R. The calculation of Hamaker constants from Lifshitz theory with applications to wetting phenomena. *Adv. Colloid Interface Sci.* **14**, 3–41 (1980).

[32] French, R. H. Origins and applications of London dispersion forces and Hamaker constants in ceramics. *J. Am. Ceram*. Soc. **83**, 2117–2146 (2000).

[33] Dagastine, R. R., Prieve, D. C. & White, L. R. Calculations of van der Waals forces in 2-dimensionally anisotropic materials and its application to carbon black. *J. Colloid Interface Sci.* **249**, 78–83 (2002).

[34] Parsegian, V. A. & Ninham, B. W. Application of the Lifshitz theory to the calculation of van der Waals forces across thin lipid films. *Nature* 224, 1197–1198 (1969).

[35] Masuda, T., Matsuki, Y. & Shimoda, T. Spectral parameters and Hamaker constants of silicon hydride compounds and organic solvents. *J. Colloid Interface Sci.* **340**, 298–305 (2009).

[36] Larruquert, J. I., Marcos, L. V. R., Mendez, J. A., Martin, P. J. & Bendavid, A. High reflectance ta-C coating in the extreme ultraviolet, *Opt. Exp.* **21**, 27537–27549 (2013).
